# ASSESSING CAREGIVING SUPPORT NETWORKS FOR PERSONS WITH DEMENTIA IN A LONG-TERM CARE CONTEXT IN CHINA AND THE US

**DOI:** 10.1093/geroni/igad104.0970

**Published:** 2023-12-21

**Authors:** Hanzhang Xu, Matthew Dupre, Scott Lynch, Truls Østbye, Bei Wu

**Affiliations:** Duke University, Durham, North Carolina, United States; Duke University, Durham, North Carolina, United States; Duke University, Durham, North Carolina, United States; Duke University School of Medicine, Durham, North Carolina, United States; New York University, New York City, New York, United States

## Abstract

Informal caregivers are vital to the provision of care for persons living with dementia (PLWD). Although PLWD often receive care from multiple sources, little is known about the broad caregiving support networks that PLWD have. Furthermore, the structure of caregiving support networks is likely influenced by the availability, accessibility, and affordability of LTC services. Therefore, caregiving support networks in China and in the U.S. are likely to differ. In this study, we identified the major types of caregiving support networks that PLWD have using the Health and Retirement Study and China Health and Retirement Longitudinal Study. Results from latent class analysis show that both countries have three major types of caregiving networks, but they differ in the proportion of each country’s population that follow each type. Unlike conventional secondary data studies that often only focus on existing data, we are working with WE-THRIVE Consortium to further gain a comprehensive understanding of the dementia care context in China and in the U.S. Through an expert panel survey, we will assess the scope, services, needs, funding, and ownerships of LTC in each country. We will then link the data from expert survey to the two national aging studies to contextualize the caregiving support networks in persons with dementia. This new way of linking existing individual-level with country-level data is directly responsive to the priority research areas identified by the National Academy of Medicine and will shed light on which caregiving support networks need more external formal support to promote person-centered dementia care.

